# Non-Pharmacological Strategies in Atherosclerotic Cardiovascular Disease: From Molecular Mechanisms to Clinical Integration

**DOI:** 10.3390/biomedicines14081868

**Published:** 2026-08-20

**Authors:** Tatyana I. Kovyanova, Maria O. Nerush, Vasily P. Karagodin, Daria D. Borodko, Ulyana V. Rozhkova, Stanislav A. Antonov, Aleksandra S. Utkina

**Affiliations:** 1 Institute for Atherosclerosis Research, 4-1-207 Osennyaya Street, 121609 Moscow, Russia; 2 Scientific and Educational Center for Molecular and Cellular Technologies, St. Petersburg Chemical Federal University, 21 Rentgen Street, 197101 St. Petersburg, Russia; 3 Institute of General Pathology and Pathophysiology, 8 Baltiiskaya Street, 125315 Moscow, Russia; 4 Laboratory of Molecular Genetic Modeling of Inflammaging, Institute of General Pathology and Pathophysiology, 8 Baltiiskaya Street, 125315 Moscow, Russia; 5 Department of Commodity Expertise and Customs Business, Plekhanov Russian University of Economics, 36 Stremyanny Lane, 115054 Moscow, Russia

**Keywords:** atherosclerotic cardiovascular disease (ASCVD), cardiometabolic risk, dietary patterns, lifestyle interventions, nutraceuticals, Omega-3 fatty acids, berberine, lipid metabolism, inflammation

## Abstract

Atherosclerotic cardiovascular disease (ASCVD) remains the leading cause of morbidity and mortality worldwide, driven by complex interactions among dyslipidemia, chronic inflammation, insulin resistance, endothelial dysfunction, and gut microbiome-derived metabolites. This review synthesizes mechanistic and clinical evidence on non-pharmacological strategies that modulate these pathways and contribute to ASCVD risk reduction. Dietary patterns such as Mediterranean, DASH, and plant-based diets improve lipid metabolism, attenuate inflammation, and enhance endothelial function. Chrononutrition approaches, including time-restricted feeding and structured fasting protocols, influence circadian regulation of glucose and lipid homeostasis. Circadian misalignment—including irregular sleep timing, shift work, and disrupted feeding–fasting cycles—independently contributes to ASCVD through impaired glucose tolerance, dyslipidemia, endothelial dysfunction, and systemic inflammation. Modulation of the gut microbiome, particularly through increased short-chain fatty acid production and reduced TMAO formation, provides additional cardiometabolic benefits. Key nutraceuticals—phytosterols, omega-3 fatty acids, and berberine—demonstrate clinically meaningful effects through micellar competition, inflammation-resolving lipid mediators, and AMPK activation, respectively. When combined with evidence-based pharmacotherapy, these interventions exert synergistic effects on cardiometabolic risk factors. This integrative biomedical framework highlights the importance of combining lifestyle, metabolic, microbiome-targeted, and nutraceutical strategies for comprehensive ASCVD prevention.

## 1. Introduction

Atherosclerotic cardiovascular disease (ASCVD) remains the leading global cause of morbidity and mortality and is now understood as a chronic inflammatory–metabolic disorder driven by endothelial injury, oxidative modification of lipoproteins, maladaptive immune activation, insulin resistance, visceral adiposity, and gut microbiome dysregulation. Despite substantial progress in LDL-lowering therapies, a considerable residual cardiovascular risk persists due to persistent inflammation, impaired HDL functionality, metabolic dysfunction, and lifestyle-related factors. These limitations have intensified interest in non-pharmacological strategies that target mechanistic pathways often unaffected by conventional drugs, including structured physical activity, defined dietary patterns, chrono-nutrition, modulation of the gut microbiome, and evidence-based nutraceuticals. This review integrates mechanistic and clinical data to outline how these interventions contribute to comprehensive ASCVD prevention and risk reduction.

## 2. The Unresolved Burden of Atherosclerosis and the Rationale for Non-Drug Interventions

**Figure 1:**
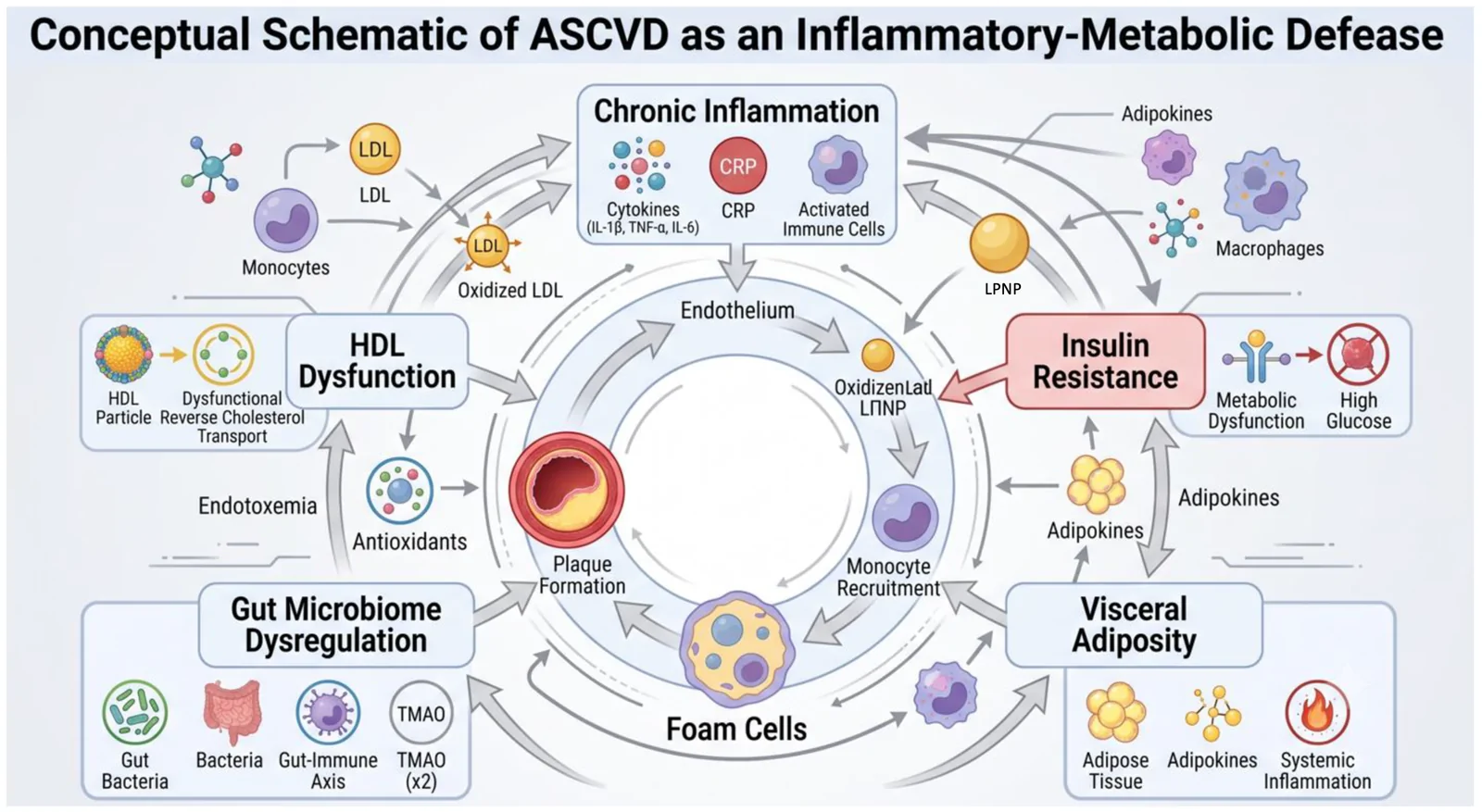
Conceptual schematic of ASCVD as an inflammatory-metabolic disease. The central cascade depicts endothelial injury leading to oxidized LDL formation, monocyte recruitment, foam cell development, and eventual plaque formation. Surrounding this core pathway are major contributors to residual cardiovascular risk—chronic inflammation, HDL dysfunction, insulin resistance, visceral adiposity, and gut microbiome dysregulation—highlighting the interconnected metabolic and immune mechanisms that sustain atherosclerosis progression.

Atherosclerotic cardiovascular disease (ASCVD) remains the single largest contributor to global morbidity and mortality, responsible for approximately 17.9 million deaths annually, with projections suggesting this figure could approach 23.6 million by 2030 [1]. According to contemporary clinical frameworks, atherosclerotic coronary artery disease (CAD) is now defined not merely as an ischemic disorder but as a chronic, progressive disease of the arterial wall driven by lipid deposition, endothelial dysfunction, inflammation, and maladaptive immune activation. Modern concepts emphasize the central role of the atheroma itself—its inflammatory activity, lipid composition, fibrous cap stability, and interaction with systemic metabolic factors—rather than focusing solely on downstream ischemia. This shift reflects the broader understanding that residual cardiovascular risk persists even after LDL-lowering, due to persistent vascular inflammation, metabolic dysfunction, and non-lipid contributors to plaque progression. The pathobiology of ASCVD is no longer viewed simplistically as a lipid-storage disorder but rather as a chronic, progressive inflammatory disease of the arterial wall, initiated by endothelial injury, propagated by oxidative modification of lipoproteins, and sustained by a maladaptive immune response involving both innate and adaptive arms. The classical paradigm—elevated low-density lipoprotein (LDL) cholesterol as the primary driver—has been enriched by the recognition that residual cardiovascular risk persists even after aggressive LDL-lowering with statins and PCSK9 inhibitors. An additional contributor to residual risk is elevated lipoprotein(a) [Lp(a)], a genetically determined LDL-like particle enriched with oxidized phospholipids. High Lp(a) levels independently increase ASCVD risk and are not substantially modified by statins or lifestyle interventions. This residual risk stems from persistent inflammation, impaired high-density lipoprotein (HDL) functionality, insulin resistance, visceral adiposity, and dysregulation of the gut microbiome. The visualization of these mechanisms is presented in Figure 1.

Pharmacological approaches, while undeniably life-saving, encounter several practical limitations: suboptimal long-term adherence (approximately 50% of patients discontinue statins within two years), side-effect profiles that limit tolerability, and the sheer cost of novel biologics, which restricts their accessibility in resource-limited settings. These realities have catalysed a resurgence of scientific and clinical interest in non-pharmacological strategies. However, contemporary approaches have moved far beyond the simplistic “eat less, move more” paradigm. The current evidence base supports a multi-layered framework that integrates structured physical activity, precisely defined dietary patterns, chrono-nutrition (meal timing and fasting protocols), and the targeted use of functional foods and nutraceuticals—substances that bridge the conventional divide between nutrition and pharmacotherapy [2]. Importantly, these interventions are not viewed as replacements for evidence-based pharmacotherapy but rather as synergistic adjuvants that address mechanistic pathways often untouched by conventional drugs.

This review synthesizes the current state of knowledge on non-pharmacological approaches for ASCVD prevention and treatment, with particular emphasis on the molecular rationale underpinning each intervention, the quality and consistency of clinical evidence, and the pragmatic challenges that must be overcome to translate these findings into routine clinical practice.

## 3. Lifestyle Interventions: Beyond Simple Recommendations

### 3.1. Physical Activity: Dose–Response, Modality, and Underlying Biology

**Figure 2:**
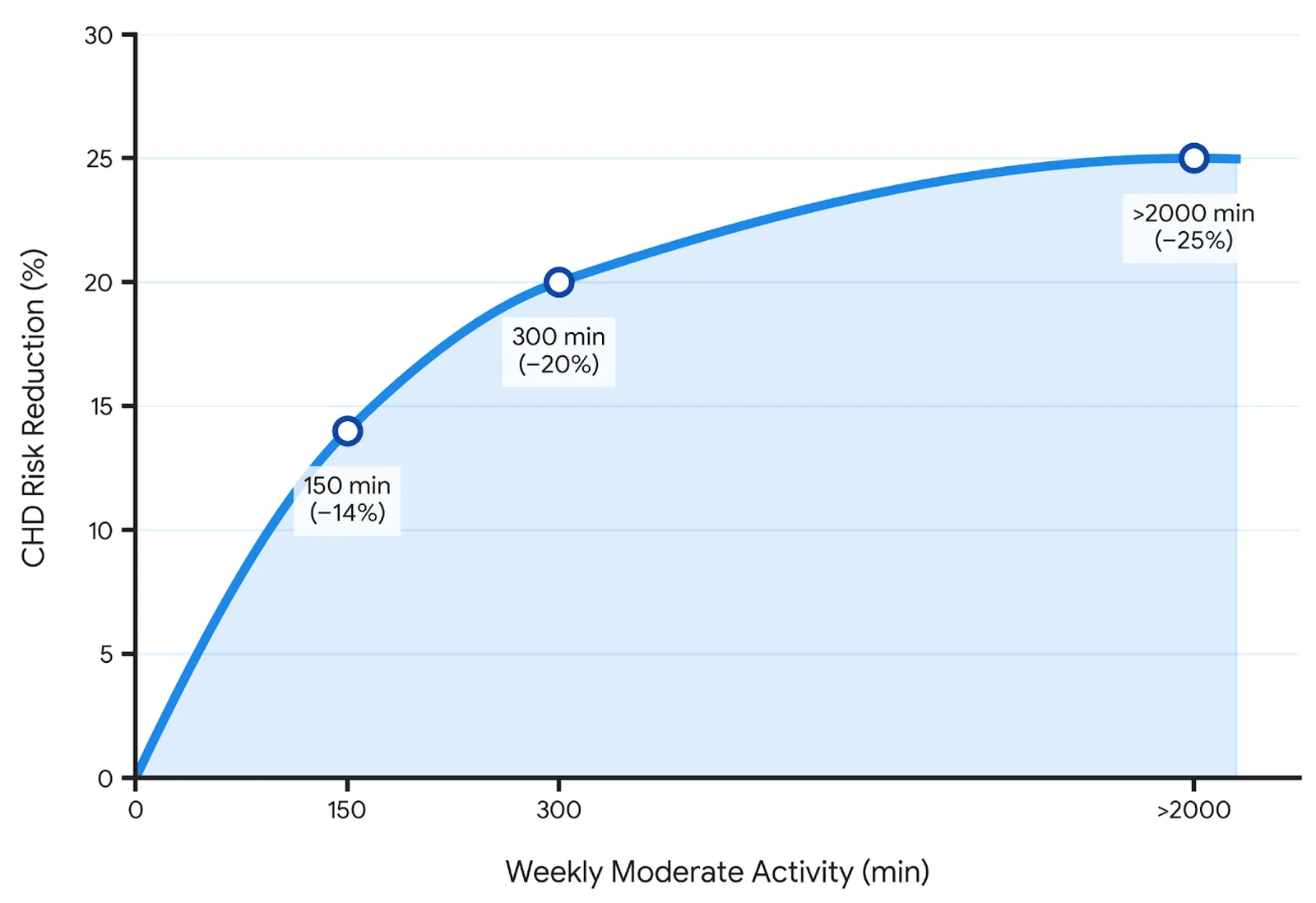
Dose–response relationship between weekly moderate physical activity and CHD risk reduction. Increasing weekly moderate-intensity activity is associated with progressive reductions in coronary heart disease risk, with the greatest relative benefit observed at lower activity volumes. Approximately 150 min per week corresponds to a ~14% reduction in CHD risk, 300 min to ~20%, and very high activity levels (>2000 min per week) to ~25%, demonstrating diminishing incremental returns at higher volumes.

The cardioprotective effects of regular physical activity have been recognised since the seminal observations of Morris and colleagues in the 1950s regarding the lower incidence of coronary events among London bus conductors compared to sedentary drivers [3]. Contemporary epidemiological evidence has refined our understanding of the dose–response relationship. A comprehensive meta-analysis of over 130,000 participants demonstrated that achieving 150 min of moderate-intensity aerobic activity per week confers a 14% risk reduction for coronary heart disease, while doubling this volume to 300 min yields a 20% reduction [4]. Notably, the curve does not plateau at the upper end; individuals exceeding 2000 min of weekly moderate activity show an approximately 25% reduction, although the incremental benefit per additional unit of effort diminishes at high volumes. The dose–response relationship between weekly moderate activity and CHD risk reduction is illustrated in Figure 2.

**Figure 3:**
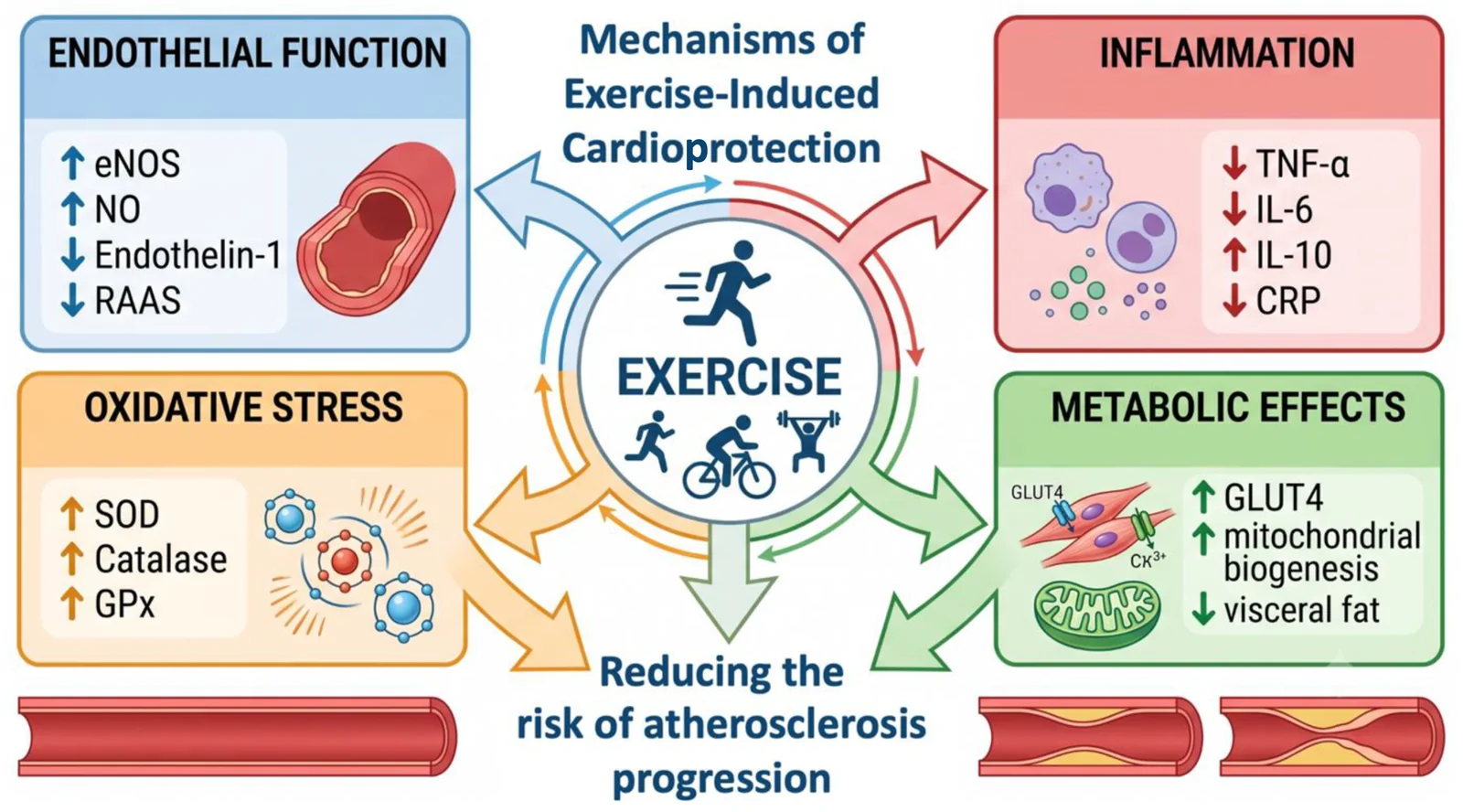
Mechanisms of exercise-induced cardioprotection. Regular physical activity improves endothelial function (↑ eNOS, ↑ nitric oxide, ↓ endothelin-1, ↓ RAAS), reduces inflammatory signaling (↓ TNF-α, ↓ IL-6, ↓ CRP), enhances antioxidant defenses (↑ superoxide dismutase, ↑ catalase, ↑ glutathione peroxidase), and promotes metabolic adaptations (↑ GLUT4 translocation, ↑ mitochondrial biogenesis, ↓ visceral adiposity). Together, these pathways contribute to reduced atherosclerosis progression and improved vascular health.

The mechanistic underpinnings of exercise-mediated atheroprotection are remarkably pleiotropic, extending far beyond the traditional focus on lipid modification. Regular aerobic training enhances endothelial function through multiple convergent pathways. The most extensively characterised mechanism involves upregulation of endothelial nitric oxide synthase (eNOS), with subsequent augmentation of nitric oxide (NO) bioavailability [5]. This vasodilatory effect is complemented by exercise-induced suppression of vasoconstrictor systems, particularly the endothelin-1 pathway and the renin–angiotensin–aldosterone axis. Beyond haemodynamic effects, physical activity exerts profound anti-inflammatory actions. Acute exercise stimulates the release of anti-inflammatory cytokines, notably interleukin-10 (IL-10) and IL-1 receptor antagonist, while suppressing the production of pro-inflammatory mediators, including tumour necrosis factor-alpha and IL-6 from adipose tissue [6]. Over time, these acute responses translate into a sustained reduction in circulating high-sensitivity C-reactive protein and other inflammatory biomarkers. Concurrently, exercise enhances the endogenous antioxidant defence system, increasing the activity of superoxide dismutase, catalase, and glutathione peroxidase, thereby mitigating oxidative stress within the vascular wall [7]. The key biological pathways through which exercise exerts cardioprotective effects are summarized in Figure 3. Beyond the pathways already discussed, exercise also activates AMPK and SIRT1, promotes PGC-1α-mediated mitochondrial biogenesis, enhances Nrf2-dependent antioxidant responses, and stimulates autophagy, collectively improving endothelial metabolism and immunometabolic regulation. These mechanisms complement eNOS activation and inflammatory suppression, providing a broader molecular rationale for exercise-induced atheroprotection.

The metabolic consequences of regular exercise include improved insulin sensitivity through enhanced GLUT4 translocation in skeletal muscle, increased mitochondrial biogenesis, and favourable modulation of adipose tissue distribution—specifically, a reduction in visceral adiposity, which is particularly pathogenic [8]. The lipid response includes modest reductions in LDL cholesterol (typically 5–10%), more substantial reductions in triglycerides (15–20%), and increases in HDL cholesterol (5–15%). Notably, the magnitude of HDL elevation correlates with baseline levels, with the greatest absolute gains observed in individuals with the lowest starting HDL concentrations [9].

### 3.2. Resistance Training: An Underappreciated Component

While aerobic activity has dominated clinical guidelines, resistance training has emerged as a complementary modality with distinct benefits. A meta-analysis of randomised controlled trials comparing combined aerobic and resistance training versus aerobic training alone demonstrated superior improvements in body composition (greater fat loss and preservation of lean mass), glycaemic control, and blood pressure in the combined group [10]. Resistance training improves vascular function through increased shear stress during muscle contraction and subsequent release of vasodilatory substances. Additionally, resistance exercise enhances the functional capacity of endothelial progenitor cells, promoting vascular repair [11]. In older adults and patients with sarcopenia—a condition increasingly recognised as a cardiovascular risk factor—resistance training may provide cardiovascular benefits that are at least comparable to moderate aerobic exercise, with the added advantage of improving physical function and reducing fall risk [12].

### 3.3. Chrono-Nutrition and Fasting Protocols

Meal timing and periodic fasting represent emerging frontiers in lifestyle-based cardiovascular prevention. Time-restricted feeding (TRF), a form of intermittent fasting that confines caloric intake to a specific window (typically 6–10 h daily), has been shown to improve multiple cardiovascular risk factors independent of calorie reduction [13]. The postulated mechanisms include enhanced autophagic clearance of damaged cellular components, upregulation of sirtuin-mediated pathways involved in longevity and metabolic regulation, and modulation of the circadian expression of metabolic genes that control lipid and glucose homeostasis [14]. An 8 h TRF protocol (eating only between 10:00 and 18:00) has been demonstrated to reduce systolic blood pressure by approximately 10 mmHg and improve insulin sensitivity in obese individuals over 12 weeks [15].

Alternate-day fasting and the 5:2 approach (very low calorie intake on two non-consecutive days per week) have also shown promise, particularly for individuals with visceral adiposity and metabolic syndrome [16]. However, the practicality of these protocols remains a barrier, with dropout rates exceeding 30% in some trials. Longer-term safety data—particularly regarding effects on bone density, nutrient adequacy, and the potential for disordered eating—remain insufficient, warranting cautious clinical implementation.

Lifestyle factors represent a central modifiable domain in ASCVD prevention, influencing inflammation, endothelial function, lipid metabolism, insulin sensitivity, and gut microbiome composition. Beyond structured physical activity, dietary patterns, and chrono-nutrition approaches discussed above, several behavioral determinants substantially shape cardiometabolic risk. Sleep regularity and circadian alignment support glucose and lipid homeostasis, while sleep disruption contributes to endothelial dysfunction and systemic inflammation. Sedentary behavior, tobacco use, excessive alcohol intake, and chronic psychological stress further amplify metabolic dysregulation and inflammatory signaling. Conversely, protective behavioral patterns—including optimal sleep duration, maintenance of regular daily rhythms, moderate habitual physical activity, and adherence to cardioprotective dietary patterns—collectively reduce residual cardiovascular risk. These behavioral determinants interact with biological risk factors such as hypertension, diabetes, visceral adiposity, and chronic inflammation, underscoring the need for integrated lifestyle strategies within ASCVD prevention frameworks.

## 4. Dietary Patterns and Their Impact on the Atherosclerotic Process

### 4.1. Mediterranean Diet: The Most Robust Evidence Base

The Mediterranean dietary pattern, characterised by high consumption of extra-virgin olive oil, nuts, legumes, whole grains, fish, and minimally processed plant foods, with moderate intake of fermented dairy and low consumption of red and processed meats, represents the most extensively validated dietary approach for ASCVD prevention [17]. The PREDIMED trial, a primary prevention study of over 7400 high-risk participants, demonstrated that a Mediterranean diet supplemented with either extra-virgin olive oil or mixed nuts significantly reduced the incidence of major cardiovascular events by 30% compared to a low-fat control diet [18]. More recently, the CORDIOPREV study confirmed the superiority of the Mediterranean diet over a low-fat diet in secondary prevention of cardiovascular disease [19]. The Lyon Heart Study, a landmark secondary prevention trial in post-myocardial infarction patients, further reinforced the cardiovascular benefits of this dietary pattern [20]. Meta-analyses and systematic reviews have consistently supported the Mediterranean diet for both primary and secondary cardiovascular prevention [21].

The protective mechanisms of the Mediterranean diet are multifaceted [22]. High intakes of monounsaturated fatty acids (primarily oleic acid) and polyunsaturated fatty acids modulate LDL particle size toward a larger, less atherogenic phenotype while increasing HDL functionality. The abundant polyphenols in olive oil, particularly hydroxytyrosol and oleuropein, exert anti-inflammatory and antioxidant effects, reducing the expression of adhesion molecules on the endothelial surface and limiting the transmigration of monocytes into the subendothelial space. The omega-3 fatty acids derived from fish intake contribute to plaque stabilisation and anti-arrhythmic effects. Furthermore, the Mediterranean diet promotes a favourable gut microbial composition, increasing the abundance of butyrate-producing bacteria that contribute to intestinal barrier integrity and systemic anti-inflammatory effects [23]. A urinary polyphenol signature of Mediterranean diet adherence, comprising compounds derived from olive oil, wine, nuts, fruits, and vegetables, has been prospectively associated with lower cardiovascular disease incidence [24].

Cardioprotective dietary patterns also modulate macrophage polarization (favoring M2 phenotypes), suppress NLRP3 inflammasome activation, preserve endothelial glycocalyx integrity, and improve mitochondrial efficiency. These mechanisms complement lipid-lowering and anti-inflammatory effects and help explain the vascular remodeling benefits observed in clinical studies.

### 4.2. DASH Diet: Blood Pressure and Beyond

The DASH diet, originally developed for hypertension management, has shown substantial cardiovascular benefits independent of its blood pressure-lowering effects [25]. This dietary pattern emphasises fruits, vegetables, whole grains, lean proteins, and low-fat dairy, while restricting sodium, saturated fat, and added sugars. The original DASH trial demonstrated that a diet rich in fruits, vegetables, and low-fat dairy products substantially reduces blood pressure [26]. Subsequent studies, including the DASH-Sodium and PREMIER trials, confirmed these effects and demonstrated the additive benefits of reduced sodium intake and comprehensive lifestyle modification [27,28]. The DASH diet consistently reduces LDL cholesterol and triglycerides, partly through increased fibre intake and reduced dietary cholesterol absorption. Furthermore, the DASH pattern has been shown to attenuate markers of endothelial dysfunction, including reductions in asymmetric dimethylarginine and improvements in flow-mediated dilation, even in normotensive individuals [29].

### 4.3. Plant-Based and Vegetarian Diets

**Figure 4:**
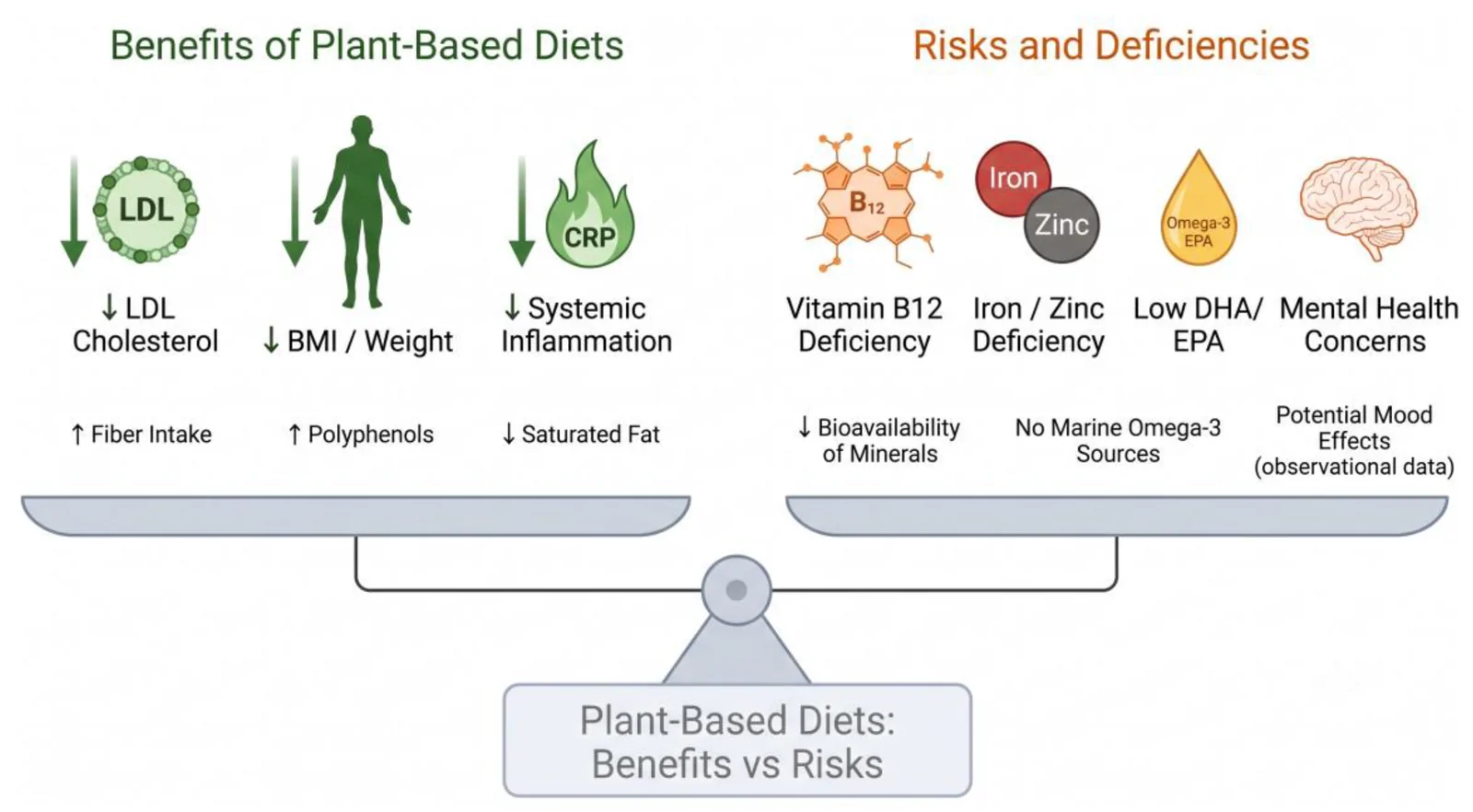
Benefits and risks of plant-based dietary patterns. Plant-based diets are associated with lower LDL cholesterol, reduced body weight, higher fiber and polyphenol intake, and decreased systemic inflammation. However, they may also lead to nutrient deficiencies, including vitamin B_12_, iron, zinc, and marine-derived omega-3 fatty acids (EPA/DHA), as well as reduced mineral bioavailability. Observational data suggest possible mood-related effects in some individuals, underscoring the importance of careful dietary planning.

Vegetarian and vegan dietary patterns have attracted interest for their potential cardiovascular benefits, although evidence quality varies [30]. The EPIC-Oxford study, a long-term prospective study, found that vegetarians had a lower risk of ischaemic heart disease compared to non-vegetarians after an average follow-up of 11.6 years [31]. Similarly, the Adventist Health Study-2 demonstrated reductions in cardiovascular disease and associated risk factors in subjects consuming vegan or vegetarian diets [32]. The benefits are mediated by lower LDL cholesterol (approximately 15% lower in vegans), lower body mass index, reduced systemic inflammation, and improved glycaemic control. However, careful attention to nutrient adequacy is required, particularly for vitamin B_12_, iron, zinc, and long-chain omega-3 fatty acids, which are less bioavailable from plant sources. Prolonged vegan diets have also been associated with mental health disorders including anxiety and depression, necessitating well-planned and balanced approaches [33]. A balanced overview of the benefits and potential risks associated with plant-based diets is presented in Figure 4.

Despite promising findings, clinical evidence remains heterogeneous. Variability in adherence, population characteristics, intervention duration, and publication bias contribute to inconsistent results across studies. Neutral or contradictory outcomes highlight the need for cautious interpretation and underscore the importance of individualized approaches.

## 5. Nutraceuticals and Bioactive Food Components: Bridging Nutrition and Pharmacotherapy

### 5.1. Phytosterols and Phytostanols: Evidence-Based Cholesterol Reduction

**Figure 5:**
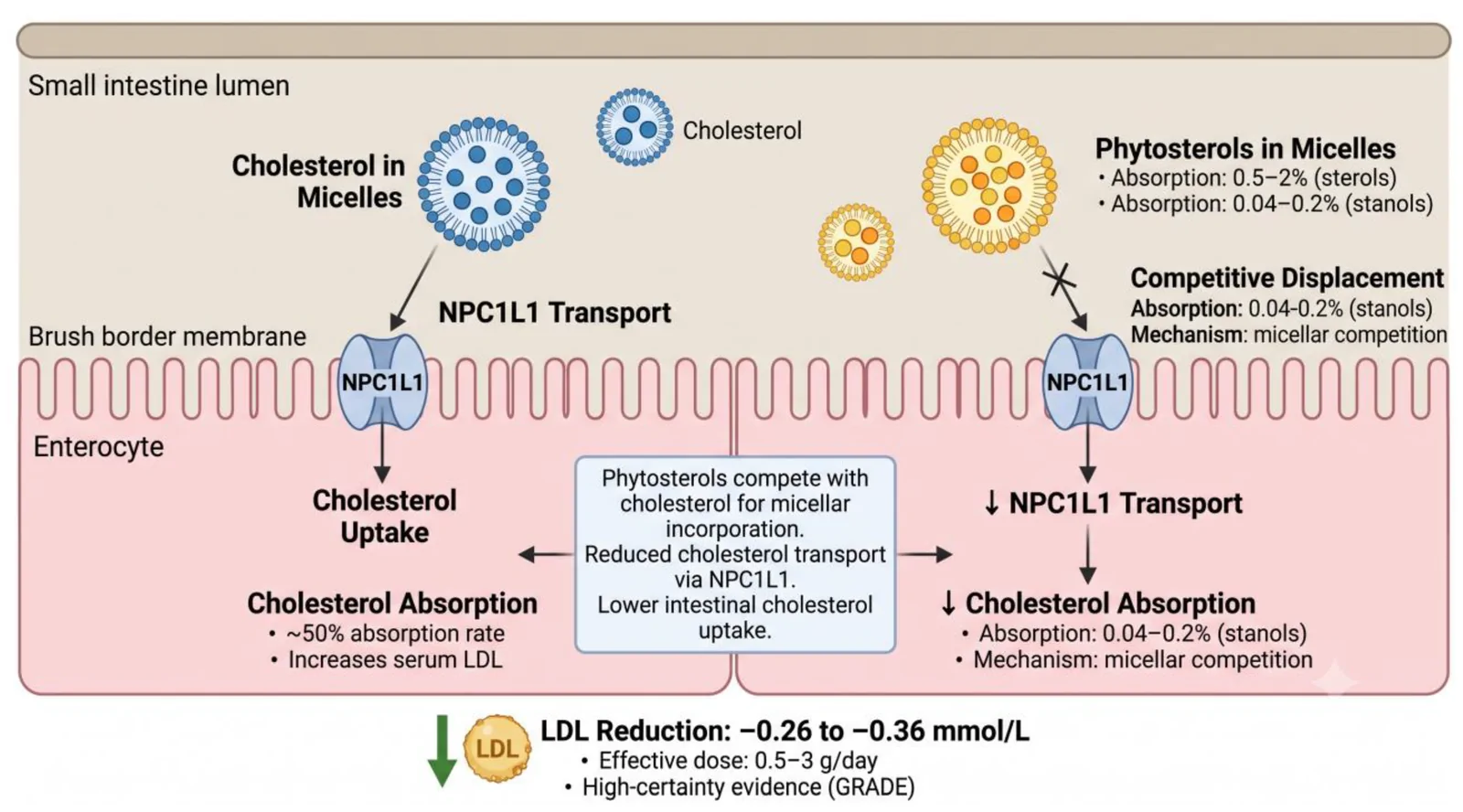
Mechanism of cholesterol and phytosterol absorption in the small intestine. Cholesterol within mixed micelles is transported across the brush-border membrane via NPC1L1, resulting in ~50% absorption and contributing to circulating LDL levels. Phytosterols and phytostanols, present in the same micelles, competitively displace cholesterol and exhibit minimal absorption (0.5–2% for sterols; 0.04–0.2% for stanols). Reduced NPC1L1–mediated uptake leads to lower intestinal cholesterol absorption and clinically meaningful LDL reductions at doses of 0.5–3 g/day.

Phytosterols (plant sterols) and their hydrogenated derivatives (stanols) represent the most extensively studied nutraceutical class for LDL cholesterol reduction. Structurally analogous to cholesterol, these compounds differ by the presence of an additional methyl or ethyl group at carbon-24, which markedly reduces their intestinal absorption (0.5–2% for sterols, 0.04–0.2% for stanols) compared to cholesterol (approximately 50%). The primary mechanism involves competitive displacement of cholesterol from mixed micelles in the intestinal lumen, thereby reducing cholesterol uptake by the Niemann–Pick C1-like 1 (NPC1L1) transporter [34] (Figure 5). This mechanism is complementary to that of statins, which act on hepatic cholesterol synthesis, enabling additive LDL reduction when phytosterols are combined with statin therapy [35].

A comprehensive umbrella review of systematic reviews and meta-analyses confirmed that phytosterols result in moderate reductions in LDL-C and total cholesterol [36]. Reductions in LDL-C ranged from −0.26 to −0.36 mmol/L across different meta-analyses, with reductions of ≥0.2 mmol/L observed at doses as low as 0.5–1 g/day [37]. Additional benefits include reductions in apolipoprotein B, high-sensitivity C-reactive protein, and systolic blood pressure, with no significant effects on HDL-C, fasting blood glucose, or HbA_1_c [38]. The certainty of evidence for LDL-C reduction was rated as high according to GRADE criteria [36]. The food matrix significantly influences efficacy; phytosterols are most effective when incorporated into fat-based matrices (margarines, spreads, yoghurts, milk) that facilitate micellar incorporation. Acceptable daily intake limits are set at 3 g per day, with no convincing evidence of serious adverse effects, although rare cases of phytosterolaemia have been reported in individuals with homozygous sitosterolaemia, a condition that contraindicates phytosterol supplementation [39].

### 5.2. Soluble Fibres and Prebiotics: Beyond Cholesterol

Beta-glucan, a viscous soluble fibre found in oats and barley, reduces LDL cholesterol through mechanisms including increased bile acid excretion, reduced cholesterol synthesis (via upregulation of hepatic LDL receptors), and decreased intestinal cholesterol absorption through increased viscosity of intestinal contents [40]. Clinical trials demonstrate an LDL reduction of approximately 5–10% with a daily intake of 3–4 g of oat beta-glucan [41]. The FDA and European Food Safety Authority (EFSA) have approved health claims linking oat beta-glucan consumption to cholesterol reduction [42]. Psyllium, a fibre derived from the *Plantago ovata* plant, provides similar effects, with meta-analyses confirming a 6–8% LDL reduction at doses of 10–15 g daily [43]. Psyllium appears to be particularly beneficial in individuals with metabolic syndrome and those with baseline LDL levels exceeding 160 mg/dL [44].

Beyond their lipid-modifying effects, soluble fibres exert prebiotic actions, increasing the abundance of beneficial gut bacteria that produce short-chain fatty acids (SCFAs), predominantly acetate, propionate, and butyrate. SCFAs act as signalling molecules, activating G protein-coupled receptors (GPR41 and GPR43) on enteroendocrine cells and adipocytes, leading to reduced appetite, increased energy expenditure, and improvements in insulin sensitivity [45]. Butyrate, in particular, enhances intestinal barrier function by increasing tight junction protein expression, thereby reducing lipopolysaccharide (LPS) translocation and subsequent systemic low-grade inflammation [46].

### 5.3. Omega-3 Polyunsaturated Fatty Acids

**Figure 6:**
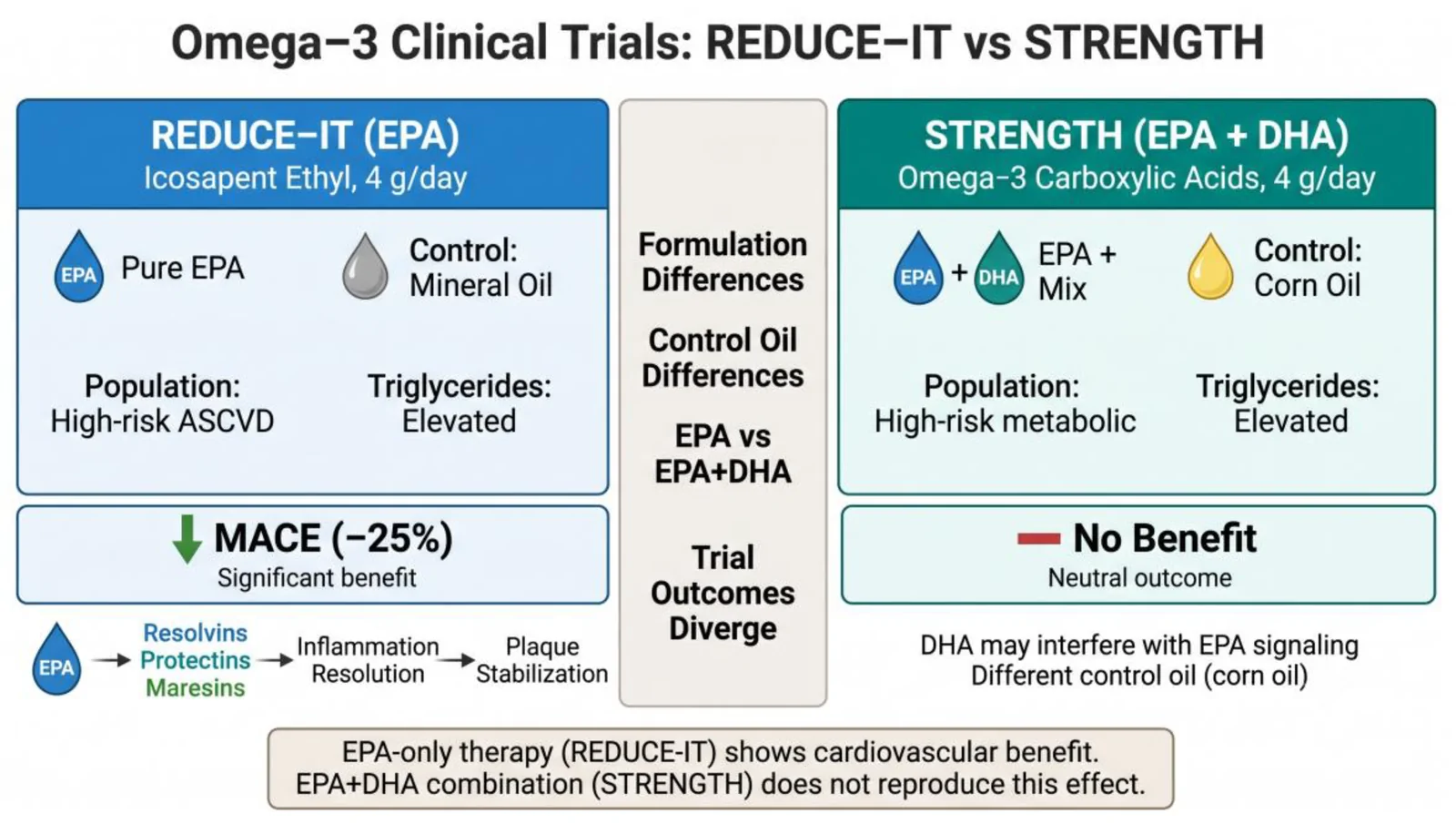
Comparison of the REDUCE-IT and STRENGTH omega-3 clinical trials. The REDUCE-IT trial evaluated high-dose purified EPA (icosapent ethyl, 4 g/day) and demonstrated a significant reduction in major adverse cardiovascular events in high-risk patients with elevated triglycerides. In contrast, the STRENGTH trial tested a combined EPA + DHA formulation (omega-3 carboxylic acids, 4 g/day) and showed no cardiovascular benefit. Key differences include the use of mineral oil versus corn oil as control, the presence of DHA in the active formulation, and potential interference with EPA-mediated inflammation-resolving pathways. These mechanistic and methodological distinctions likely contribute to the divergent clinical outcomes.

Eicosapentaenoic acid (EPA) and docosahexaenoic acid (DHA), the long-chain omega-3 fatty acids primarily derived from marine sources, have demonstrated consistent triglyceride-lowering effects (approximately 25–30% reduction at a dose of 3–4 g daily) and dose-dependent anti-inflammatory actions [47]. The REDUCE-IT trial, which used a high-dose formulation of purified EPA (icosapent ethyl, 4 g daily) in patients with elevated triglycerides and established cardiovascular disease, reported a 25% reduction in major adverse cardiovascular events [48]. Conversely, the STRENGTH trial, which evaluated a carboxylic acid formulation of both EPA and DHA, showed no such benefit [49]. The discrepancy between these trials has generated substantial discussion, with potential explanations including differences in control oils (mineral oil in REDUCE-IT versus corn oil in STRENGTH), the potential interference of DHA with EPA’s effects, and differences in baseline characteristics of study populations [50]. A comparative overview of the REDUCE-IT and STRENGTH trials is presented in Figure 6.

Meta-analyses of marine n-3 fatty acids demonstrate significant reductions in myocardial infarction (rate ratio 0.88, 95% CI 0.83–0.94), CHD death (0.92, 0.86–0.98), and total cardiovascular disease (0.95, 0.82–0.98), though these effects are attenuated but remain significant when REDUCE-IT data are removed from the analysis [51].

The cardioprotective actions of omega-3 fatty acids extend beyond lipid modification. EPA and DHA incorporate into membrane phospholipids, altering membrane fluidity and influencing the formation of specialized pro-resolving mediators (SPMs), including resolvins, protectins, and maresins, which actively resolve inflammation rather than simply suppressing it [52]. Additionally, omega-3 fatty acids stabilise atherosclerotic plaques, reduce platelet aggregation, and improve endothelial function through increased nitric oxide bioavailability [53]. Nonetheless, the clinical benefits appear to be dose-dependent and may be attenuated at lower intakes (less than 1 g daily), and the evidence for dietary fish consumption (two servings of oily fish weekly) is stronger than for over-the-counter fish oil supplements, which may have variable EPA/DHA content and oxidative stability [54].

### 5.4. Berberine: A Unique Nutraceutical with Multiple Actions

**Figure 7:**
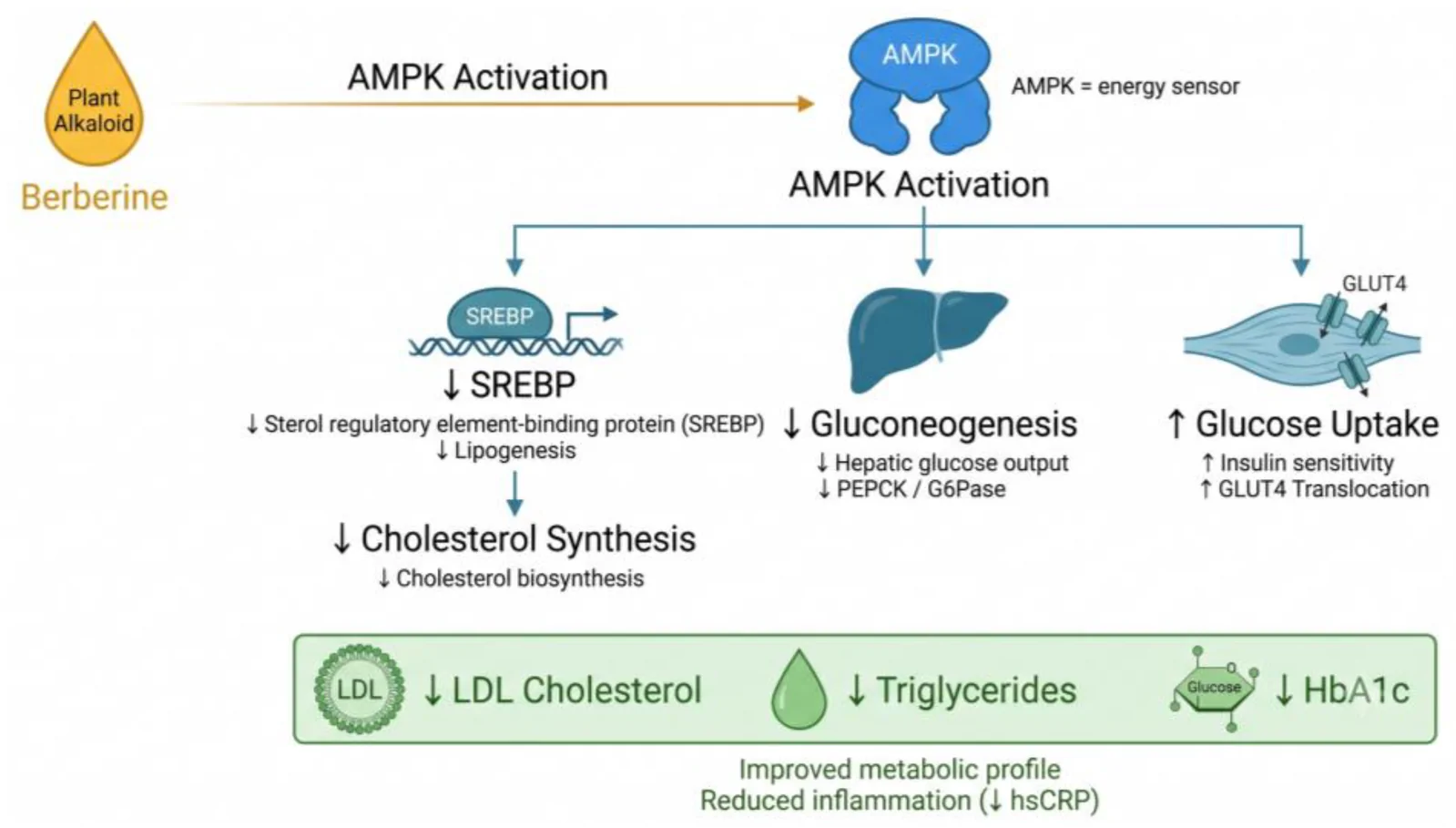
AMPK-mediated mechanisms of berberine’s metabolic and lipid-lowering effects. Berberine activates AMPK, leading to suppression of SREBP-driven lipogenesis and reduced cholesterol synthesis, inhibition of hepatic gluconeogenesis (↓ PEPCK, ↓ G6Pase), and enhanced peripheral glucose uptake through increased GLUT4 translocation. These coordinated actions result in reductions in LDL cholesterol, triglycerides, and HbA1c, alongside improvements in insulin sensitivity and systemic inflammation (↓ hsCRP).

Berberine, an isoquinoline alkaloid derived from plants such as *Coptis chinensis* and *Berberis aristata*, has emerged as one of the most promising nutraceuticals for metabolic and cardiovascular health. Berberine activates AMP-activated protein kinase (AMPK), a critical sensor of cellular energy status, leading to reduced hepatic gluconeogenesis, increased peripheral glucose uptake, and inhibition of cholesterol synthesis through downregulation of the sterol regulatory element-binding protein (SREBP) pathway [55]. These actions translate into clinically meaningful reductions in fasting glucose (approximately 20–30 mg/dL), glycated haemoglobin (0.5–1.0%), LDL cholesterol (15–20 mg/dL), and triglycerides (20–30 mg/dL) [56]. Importantly, berberine improves endothelial function and reduces inflammatory markers, including hs-CRP and TNF-α [57]. The molecular pathways through which berberine exerts its metabolic effects are summarized in Figure 7.

A meta-analysis of randomized controlled trials is currently underway to evaluate the effect of berberine on components of metabolic syndrome, including blood pressure, triglycerides, blood glucose, and waist circumference [58]. A randomized controlled trial in patients with antipsychotic-associated metabolic syndrome demonstrated that berberine (600 mg/d) significantly reduced weight, body mass index, total cholesterol, and LDL cholesterol compared to placebo over 12 weeks [59]. The effect sizes were substantial: mean differences of −0.58 mmol/L for total cholesterol (d = 1.31) and −0.52 mmol/L for LDL cholesterol (d = 1.19) [59]. Berberine’s bioavailability is limited by extensive first-pass metabolism and efflux by P-glycoprotein, but dose-finding studies have established an effective dose of 500 mg two to three times daily (total 1000–1500 mg daily) [60]. Mild gastrointestinal side effects (diarrhoea, constipation, abdominal bloating) are common but typically transient, with dose titration improving tolerability. Long-term safety data beyond two years are limited.

### 5.5. Flavonoids

Flavonoids, a major subclass of dietary polyphenols naturally present in fruits, vegetables, tea, cocoa, and olive oil, also contribute to atheroprotection through mechanisms consistent with the broader polyphenol effects described earlier. These compounds enhance endothelial nitric oxide bioavailability, reduce oxidative stress, and attenuate inflammatory signaling pathways involved in plaque progression. Flavonoid-rich foods are integral components of Mediterranean and plant-based dietary patterns discussed above, and their vascular benefits are considered part of the overall cardioprotective effects attributed to polyphenol intake.

## 6. Gut Microbiome Modulation: A New Frontier in Cardiovascular Health

The gut microbiome has emerged as a key intermediary between dietary intake and cardiovascular risk. The composition of the gut microbiota influences the metabolism of dietary components, producing metabolites that can either promote or protect against atherosclerosis [61]. The most extensively studied microbial metabolite is trimethylamine N-oxide (TMAO). Dietary phosphatidylcholine (lecithin), choline, and L-carnitine—found in red meat, eggs, and certain fish—are metabolized by gut bacteria to trimethylamine (TMA), which is subsequently oxidized by hepatic flavin-containing monooxygenase-3 (FMO3) to TMAO [62]. Elevated plasma TMAO concentrations are independently associated with incident cardiovascular events, including myocardial infarction, stroke, and cardiovascular death, in multiple large-scale cohort studies [63].

TMAO promotes atherosclerosis through multiple mechanisms: it enhances the expression of scavenger receptors (CD36 and SR-A1) on macrophages, increasing foam cell formation; it reduces reverse cholesterol transport by downregulating ABCA1 and ABCG1; it activates the inflammasome, promoting vascular inflammation; and it impairs endothelial function through reduced nitric oxide bioavailability and increased expression of adhesion molecules [64].

Targeting the gut microbiota to reduce TMAO production represents a potential non-pharmacological strategy for cardiovascular risk reduction. Resveratrol, berberine, and certain polyphenols have been shown to suppress TMAO production in animal models by altering gut microbial composition and reducing the abundance of TMA-producing bacteria [65]. In human studies, Mediterranean dietary patterns and high-fibre diets have been associated with lower TMAO levels, likely due to shifts in the gut microbial community toward a less pro-inflammatory composition [66].

Conversely, dietary fibre and polyphenols promote the production of SCFAs (acetate, propionate, butyrate) through microbial fermentation. SCFAs, particularly butyrate, exert multiple protective effects, including enhancement of gut barrier function, reduction in systemic inflammation through GPR-mediated signaling, and improvement of insulin sensitivity [67]. High-SCFA-producing microbial communities, enriched by dietary fibre (≥25–35 g daily), have been associated with lower blood pressure, reduced inflammatory markers, and improved metabolic profiles [68].

## 7. Traditional Medicine Approaches and Integrative Frameworks

Traditional medical systems offer distinct conceptual frameworks for cardiovascular health. Ayurveda, the traditional healthcare system of India, views atherosclerosis as a disorder of “Abaddha Medas” (abnormal fat) and “Dhamani Pratichaya” (vascular thickening), with descriptions of “Kaphaja Hridroga” that can be correlated to ASCVD [69]. Ayurveda management principles give more emphasis on prevention and non-pharmacological approaches such as diet and lifestyle modifications, which can be tactfully integrated into conventional therapy for providing more wholesome care [70].

Integrative frameworks, developed to facilitate the implementation of non-pharmacological interventions, emphasise multidisciplinary care teams, rigorous assessment, and the development of individualised plans that integrate traditional modalities with evidence-based lifestyle recommendations and pharmacotherapy. These frameworks are particularly relevant in settings where traditional medicine remains culturally embedded and widely accepted [71].

Traditional systems such as Ayurveda can be conceptually integrated with modern molecular frameworks through shared themes of inflammation reduction, metabolic balance, and gut–immune axis modulation. However, clinical evidence remains limited, and these approaches should be interpreted within an evidence-based context.

## 8. Clinical Integration, Evidence Gaps, and Future Directions

### 8.1. Translating Evidence into Practice

Despite the substantial evidence supporting non-pharmacological interventions, translation into routine clinical practice remains suboptimal. Barriers include limited time during clinical consultations, insufficient physician training in lifestyle counseling, and the complexity of motivating behavioural change [72]. Integration of non-physician healthcare professionals—dietitians, exercise physiologists, health coaches—into cardiovascular care teams is essential to overcome these barriers. Brief structured interventions, such as the “5As” framework (Ask, Advise, Assess, Assist, Arrange), have been validated for smoking cessation and can be adapted for lifestyle counseling [73]. Digital health interventions, including smartphone applications and wearable activity monitors, offer scalable approaches to support behaviour change, though their long-term effectiveness remains uncertain and requires further validation [74].

### 8.2. Evidence Quality and Standardization

One of the greatest challenges in the nutraceutical field is the substantial heterogeneity in product quality, bioavailability, and dosing. Over-the-counter supplements are not regulated with the same rigour as pharmaceutical products, and independent analyses have consistently revealed variability in active ingredient content, with some products failing to meet label claims [75]. For example, analyses of fish oil supplements have demonstrated EPA/DHA levels ranging from 30–80% of label claims, and oxidation markers vary widely [76]. Similarly, plant sterol formulations vary in esterification and matrix, influencing efficacy [36].

Another significant limitation is that the majority of studies have examined single compounds in isolation, using purified extracts that may not reflect the effects of whole foods. Evidence from whole-food dietary patterns (Mediterranean diet, DASH) suggests synergistic effects that are greater than the sum of individual components, likely due to additive or complementary mechanisms. Future research should move toward studying complex dietary patterns and whole-food sources of bioactive compounds, rather than isolated nutraceuticals, to better replicate real-world dietary scenarios [77].

### 8.3. Personalized Approaches

Genetic polymorphisms influence the response to both lifestyle interventions and nutraceuticals. Variation in genes encoding apolipoprotein E (*APOE*), lipoprotein lipase (*LPL*), and the LDL receptor affect lipid responses to dietary interventions [78]. Polymorphisms in the *FTO* gene influence the extent of weight loss with exercise and dietary modification, and variants in the *TAS2R38* bitter taste receptor may determine dietary fat preferences [79]. Future non-pharmacological strategies will increasingly incorporate genetic, metabolomic, and microbiome profiling to tailor interventions for maximum efficacy and adherence, moving toward a precision medicine approach [80].

Emerging precision medicine tools—including multi-omics profiling, nutrigenomics, microbiome-based dietary personalization, metabolomic signatures, and AI-assisted behavioral interventions—are expected to transform lifestyle-based ASCVD prevention. Wearable devices and digital health platforms further enable continuous monitoring of metabolic responses, facilitating individualized and adaptive lifestyle prescriptions.

### 8.4. Safety and Drug-Nutrient Interactions

Nutraceuticals are not risk-free. Phytosterols may reduce absorption of fat-soluble vitamins, including β-carotene, vitamin E, and lycopene, particularly at high doses, requiring monitoring and possible adjustment of dietary intake of these nutrients [80]. Omega-3 fatty acids at high doses (≥4 g daily) may increase the risk of bleeding, particularly in patients on concurrent antiplatelet or anticoagulant therapy, and careful monitoring is required [80]. Berberine may inhibit CYP2D6 and CYP3A4 enzymes, potentially affecting the metabolism of medications such as statins and certain antihypertensives, and it may also interact with digoxin through inhibition of P-glycoprotein [60]. Clinicians should routinely inquire about nutraceutical use and evaluate potential interactions as part of comprehensive medication reconciliation.

## 9. Discussion

Non-pharmacological strategies for ASCVD prevention converge on several shared biological pathways, including endothelial dysfunction, chronic inflammation, oxidative stress, mitochondrial impairment, and immunometabolic dysregulation. Exercise activates eNOS, reduces inflammatory cytokines, and enhances antioxidant defenses, but also engages broader metabolic regulators such as AMPK, SIRT1, PGC-1α, Nrf2, and autophagy, supporting endothelial resilience and mitochondrial efficiency. Dietary patterns—including Mediterranean, DASH, plant-based, and time-restricted feeding—modulate lipid metabolism, macrophage polarization, NLRP3 inflammasome activity, endothelial glycocalyx integrity, and gut microbiome composition, providing mechanistic explanations for their vascular benefits beyond traditional lipid-lowering effects.

Despite these promising mechanisms, clinical evidence remains heterogeneous across interventions. Variability in adherence, population characteristics, intervention duration, and publication bias contributes to inconsistent findings in intermittent fasting, plant-based diets, and nutraceutical trials. The discrepant outcomes of REDUCE-IT and STRENGTH illustrate how differences in placebo oils, EPA formulation, achieved plasma concentrations, and baseline metabolic profiles can influence results. These uncertainties highlight the need for cautious interpretation and individualized application of lifestyle interventions.

Evidence hierarchy also varies substantially. Aerobic exercise, Mediterranean diet, and DASH diet are supported by high-quality randomized trials and are incorporated into contemporary ESC and AHA/ACC guidelines. In contrast, intermittent fasting, microbiome-targeted approaches, and most nutraceuticals remain investigational, with mixed evidence from smaller trials and observational studies. Distinguishing guideline-supported interventions from emerging strategies is essential for clinical translation.

Precision medicine approaches are likely to reshape lifestyle-based ASCVD prevention. Multi-omics profiling, nutrigenomics, microbiome-derived metabolic signatures, and digital health technologies may enable individualized dietary and behavioral prescriptions. These tools can help identify patients who respond preferentially to specific dietary patterns, fasting regimens, or microbiome-modulating interventions, enhancing the effectiveness of non-pharmacological strategies.

Gut microbiome mechanisms extend beyond TMAO and short-chain fatty acids. Bile acid metabolism via FXR and TGR5, microbial tryptophan metabolites, intestinal permeability, endotoxemia, and host–microbiome metabolic interactions represent additional pathways linking lifestyle factors to vascular inflammation and metabolic dysfunction. Integrating these emerging concepts provides a more comprehensive understanding of microbiome–cardiovascular interactions.

Finally, traditional medicine approaches such as Ayurveda may conceptually align with modern molecular frameworks through shared themes of inflammation reduction, metabolic balance, and gut–immune modulation. However, clinical evidence remains limited, and these approaches should be interpreted within an evidence-based context to maintain coherence with contemporary cardiovascular prevention strategies.

## 10. Limitations

This review has several limitations that should be acknowledged when interpreting the findings. First, the included evidence is heterogeneous, spanning randomized controlled trials, observational cohorts, mechanistic studies, and narrative reports, with substantial variability in study quality, follow-up duration, and population characteristics. These differences limit direct comparability across interventions and may contribute to inconsistent or neutral findings reported in fasting protocols, plant-based diets, and nutraceutical trials. Second, many mechanistic insights—particularly those related to immunometabolism, mitochondrial biology, and gut microbiome signaling—are derived from preclinical or short-term human studies, and their long-term clinical relevance remains uncertain. Third, adherence to lifestyle interventions is highly variable and often poorly quantified, reducing the generalizability of trial outcomes to real-world settings. Fourth, several emerging areas, including microbiome-targeted therapies, precision nutrition, and multi-omics-guided personalization, are supported primarily by early-phase or exploratory studies published in 2025–2026, and robust clinical validation is still lacking. Fifth, publication bias may favor positive lifestyle intervention studies, while neutral or negative results remain underreported. Finally, traditional medicine approaches such as Ayurveda have limited high-quality evidence, and their integration into contemporary cardiovascular prevention frameworks requires cautious interpretation.

## 11. Conclusions

Non-pharmacological approaches for the prevention and treatment of atherosclerotic cardiovascular diseases have evolved from general, non-specific recommendations to precisely defined, mechanistically informed strategies. Regular physical activity, encompassing both aerobic and resistance training, provides multi-organ benefits through improved endothelial function, reduced inflammation, metabolic optimisation, and enhanced vascular repair. Dietary patterns—Mediterranean, DASH, and plant-based—confer substantial cardiovascular benefits that extend beyond traditional risk factor modification, with the gut microbiome emerging as a critical mediator of dietary effects. Nutraceuticals and functional foods, including phytosterols, beta-glucan, omega-3 fatty acids, and berberine, offer evidence-based adjunctive benefits for lipid and metabolic management, while polyphenols and other bioactive compounds provide pleiotropic mechanisms with considerable promise, though bioavailability remains a key barrier to clinical translation.

**Figure 8:**
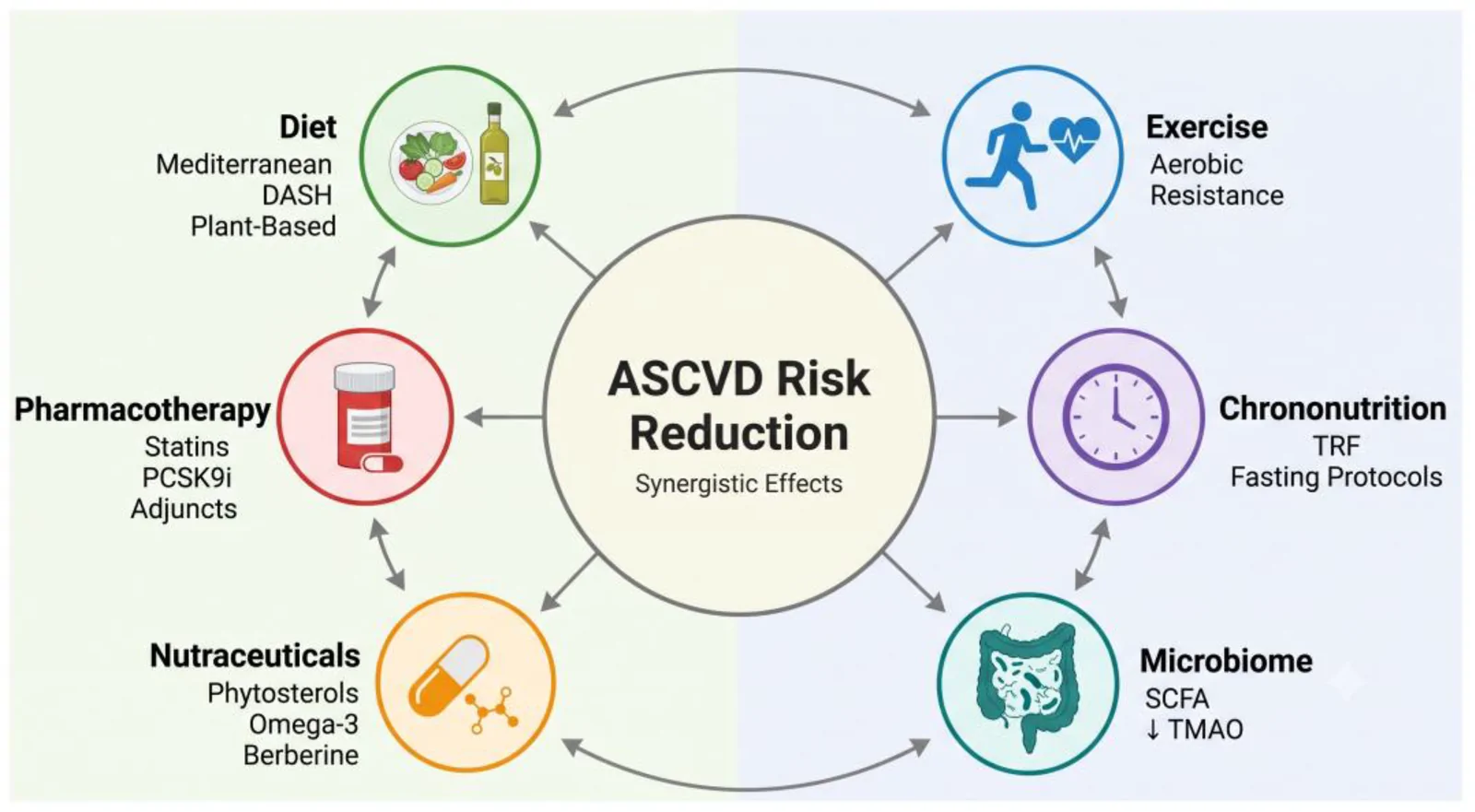
Synergistic framework for ASCVD risk reduction across lifestyle, metabolic, microbiome, nutraceutical, and pharmacological domains. Multiple evidence-based interventions contribute to ASCVD risk reduction through complementary mechanisms. Dietary patterns (Mediterranean, DASH, plant-based), structured exercise (aerobic and resistance training), chrononutrition approaches (time-restricted feeding and fasting protocols), gut microbiome modulation (↑ SCFA, ↓ TMAO), nutraceuticals (phytosterols, omega-3 fatty acids, berberine), and pharmacotherapy (statins, PCSK9 inhibitors, adjunctive agents) interact to produce additive and synergistic cardioprotective effects. This integrative model highlights the importance of combining lifestyle and medical strategies for optimal cardiovascular prevention.

The integration of these approaches requires a shift from a disease-oriented, pharmacological paradigm to a holistic, person-centred framework that acknowledges the contributions of diet, physical activity, chrono-nutrition, and gut microbial health. This paradigm shift demands multidisciplinary care teams, robust patient education, and ongoing support for behaviour change. The future of cardiovascular prevention lies not in choosing between pharmacological and non-pharmacological strategies, but in strategically combining them in a personalised manner that addresses the unique risk factor profile, genetic background, and lifestyle context of each patient. Achieving this integration will require continued research investment, regulatory reforms to ensure nutraceutical quality and safety, and healthcare system redesign to prioritise preventive, lifestyle-based interventions alongside evidence-based pharmacotherapy. The multidimensional and synergistic nature of ASCVD risk reduction strategies is illustrated in Figure 8.

Collectively, exercise, dietary interventions, microbiome modulation, nutraceuticals, and pharmacotherapy converge on shared biological pathways—including endothelial dysfunction, chronic inflammation, oxidative stress, mitochondrial impairment, and immunometabolic dysregulation. Integrating these mechanistic insights provides a unified framework for comprehensive ASCVD prevention.

## Data Availability

No new data were created or analyzed in this study. Data sharing is not applicable to this article.
